# Pathogenesis of the Metabolic Syndrome: Insights from Monogenic Disorders

**DOI:** 10.1155/2013/920214

**Published:** 2013-05-21

**Authors:** Rinki Murphy, Richard W. Carroll, Jeremy D. Krebs

**Affiliations:** ^1^Department of Medicine, Faculty of Medical and Health Sciences, University of Auckland, Private Bag 92019, Auckland, New Zealand; ^2^Endocrine, Diabetes and Research Centre, Wellington Regional Hospital, Private Bag 7902, Newtown, Wellington 6021, New Zealand; ^3^Department of Medicine, University of Otago, Newtown, Wellington 6021, New Zealand

## Abstract

Identifying rare human metabolic disorders that result from a single-gene defect has not only enabled improved diagnostic and clinical management of such patients, but also has resulted in key biological insights into the pathophysiology of the increasingly prevalent metabolic syndrome. Insulin resistance and type 2 diabetes are linked to obesity and driven by excess caloric intake and reduced physical activity. However, key events in the causation of the metabolic syndrome are difficult to disentangle from compensatory effects and epiphenomena. This review provides an overview of three types of human monogenic disorders that result in (1) severe, non-syndromic obesity, (2) pancreatic beta cell forms of early-onset diabetes, and (3) severe insulin resistance. In these patients with single-gene defects causing their exaggerated metabolic disorder, the primary defect is known. The lessons they provide for current understanding of the molecular pathogenesis of the common metabolic syndrome are highlighted.

## 1. Introduction

Insulin resistance is implicated in the pathophysiology of the twin epidemics of type 2 diabetes and obesity. Both conditions are associated with a high burden of premature morbidity and mortality globally; however, the dependence of one on the other is not complete. Despite intensive efforts, the molecular mechanisms underlying the relationship between obesity, insulin resistance, and type 2 diabetes in the metabolic syndrome are incompletely understood. 

The accelerating discovery of single-gene defects resulting in rare types of diabetes, obesity, or severe insulin resistance over the past 20 years provides the opportunity to unravel the role of several key mediators in these separate groups of disorders through firmer cause-effect relationships. We summarise the key monogenic disorders that result in nonsyndromic obesity, pancreatic beta-cell diabetes, or severe insulin resistance and discuss how the insights they provide may be applied to the understanding of more prevalent metabolic disease (see summary Table 1).

## 2. Monogenic Nonsyndromic Obesity

**Figure 1:**
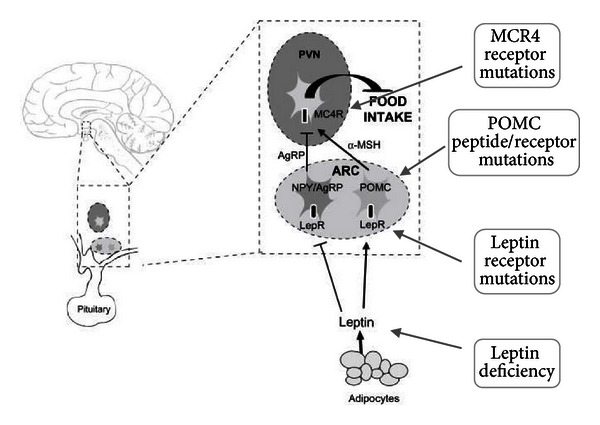
Diagram showing the sites of known monogenic causes of obesity which affect the central regulators of appetite. Leptin is one of the major adiposity signals which circulates to the brain in the region of the arcuate nucleus (ARC) within the hypothalamus and binds to its receptors located in two groups of ARC neurons. Leptin action promotes the synthesis of proopiomelanocortin (POMC) which is cleaved to *α*-melanocyte stimulating hormone (*α*MSH), a neurotransmitter which acts at melanocortin 4 receptors (MC4R) on neurons in other hypothalamic areas to reduce food intake. Leptin acts to inhibit the synthesis and secretion of Agouti-related peptide (AgRP) from the second group of ARC neurons, which is an antagonist at MC4R.

*Lesson 1: Proof That Humans Can Become Obese as a Result of Single-Gene Defects Controlling Key Central Components of Appetite.* At a very fundamental level, obesity is the result of excess energy intake over energy expenditure over a prolonged period of time. The rapid increase in worldwide rates of overweight and obesity over the last 30–40 years suggests a predominant change in environmental, diet, and lifestyle factors rather than any change in genetics as the main cause of the obesity epidemic. However, it is clear that there are important genetic contributions to the susceptibility to becoming obese and to the associated comorbidities. Over the last 2 decades, there has been an increased understanding of mechanisms controlling energy balance and appetite regulation in particular. Much of this has come about through the discovery of genes responsible for appetite regulation (see Figure 1). These genes have been identified by characterization of genetic variants of candidate genes in severely obese rodent models and subsequently humans.

### 2.1. Leptin and Leptin Receptor Mutations

Leptin was one of the earliest hormones involved in energy balance to be identified [17] and found to be the responsible factor deficient in the severely obese ObOb mouse model of obesity. Leptin is released from adipocytes in proportion to adipose tissue mass and circulating levels are greater in women than men [18]. Leptin has many biological roles including effects on reproduction, bone mineral density, and the immune system; however, one of the main functions is in appetite regulation by signaling of adipose stores by binding to leptin receptors in the arcuate nucleus of the hypothalamus [19].

The downstream effect of leptin receptor activation is regulation of neuropeptides such as those from the proopiomelanocortin (POMC) and agouti-related peptide (AGRP) neurons. Leptin downregulates orexigenic (appetite promoting) neuropeptides such as neuropeptide Y (NPY) and agouti-related peptide (AGRP) but also upregulates anorexigenic neuropeptides such as *α* melanocyte stimulating hormone (*α*MSH) and cocaine and amphetamine-regulated transcripts (CART) [20]. It might therefore be predicted that circulating leptin concentrations would be reduced in those with obesity, therefore resulting in inadequate appetite regulation. However, leptin levels are increased in common obesity in proportion to excess fat mass [21], raising the possibility of so-called “leptin resistance”. 

Leptin also has an important reproductive function. The development of adequate adipose stores to allow puberty to begin is signalled by leptin to the hypothalamus. Thus leptin has a permissive effect on puberty [22]. Furthermore, if adipose stores reduce and leptin levels fall, gonadotrophin levels and pulsatility are affected such that ovulation is inhibited [22]. 

Evidence for the important role of leptin in energy regulation in humans comes from the observations of O'Rahilly et al., who identified children with severe early onset of obesity who had undetectable levels of leptin [1]. They were found to be homozygous for a frameshift mutation in the *leptin* gene which resulted in a truncated protein which was not secreted. They were morbidly obese with excess fat mass, hyperphagia, but no changes in resting metabolic rate or total energy expenditure adjusted for body composition. These children were the human equivalent of the obese (ob/ob) mouse, resulting from a recessive homozygous mutation in the *leptin* gene and confirmed a critical role for leptin in human appetite regulation. This was reinforced by the observation that replacement of recombinant human leptin in these children resulted in a rapid reversal of the hyperphagia, promoted weight loss, and normalization of body composition [23]. Further support for the importance of leptin comes from identification of a leptin receptor mutation and characterization of humans with homozygous loss of leptin receptor function [2]. These individuals have very high circulating leptin concentrations but a very similar phenotype to those with leptin deficiency. However, as would be predicted, they do not respond to treatment with additional leptin.

### 2.2. Proopiomelanocortin Gene Mutations

Further evidence for the central role of appetite regulation comes from other monogenic defects of the *POMC* gene, which codes for a number of important proteins including adrenocorticotrophin hormone (ACTH) and melanocortin peptides (*α* and *β* melanocyte stimulating hormone (MSH)). Individuals have been identified with homozygous mutations of the *POMC* gene resulting in complete loss of function of all POMC-derived peptides [24]. They present early in life with hypocortisolemia secondary to ACTH deficiency. Obesity with hyperphagia develops even with cortisol deficiency but becomes accelerated after cortisol replacement. 

Further insights are provided by mutations of specific POMC-derived peptides. Point mutations that disrupt the *α*-MSH or *β*-MSH peptides have been linked with early onset of human obesity depending on the location of the mutation [3, 25, 26].

### 2.3. Melanocortin Receptor Mutations

The importance of the melanocortin peptides is confirmed by the demonstration of severe obesity associated with mutations of the melanocortin receptor (*MC4R*). The melanocortin receptor MC4R is expressed on neurons in the paraventricular nucleus of the hypothalamus. Activation of MC4R results in release of anorexigenic peptides such as brain-derived neurotrophic factor (BDNF) [4]. Mutations of *MC4R* are found in up to 6% of severe childhood obesity [27, 28]. Whilst obesity appears to be dominantly inherited, the penetrance of obesity with *MC4R* mutations is variable with mutations resulting in complete loss of function having a more severe phenotype. This is demonstrated by the variation in the degree of hyperphagia with ad libitum energy intake. Those with partial *MC4R* deficiency have less excess intake compared with those with complete deficiency [27]. This relationship between degree of function of the mutant receptors and energy intake highlights the importance of the *MC4R* in energy balance. 

However, the complexity of the system and the interrelationship of the signals are also demonstrated by the variability in the ad libitum energy intake seen when comparing those with leptin deficiency, inactive and partially active *MC4R* mutations. Those with leptin deficiency have the greatest energy intake, those with inactive MC4R mutations are less hyperphagic, and those with partially active mutations have similar intake to those with treated leptin deficiency [27].

The discovery of peptides and receptors critical to the pathways of energy balance opens the opportunity for therapeutic targets for common obesity. This is demonstrated by the effectiveness of the replacement of leptin in those with leptin deficiency [23]. However, in common obesity, where circulating leptin levels are already high, supplemental leptin therapy has little additional effect on appetite or fat mass [29]. To date no therapeutic agents acting on the other peptides or receptors described have been successfully developed.

### 2.4. Brain-Derived Neurotrophic Factor and Receptor Mutations

Other players in the central control of appetite have also been identified through rare genetic mutations resulting in reduced production of peptides such as brain-derived neurotrophic factor (BDNF) [5] or loss of function of receptors such as the BDNF receptor tropomyosin-related kinase B (*TrkB*) [6]. Both mutations result in hyperphagia and obesity. These mutations also highlight the pleiotropic function of these peptides and the activation of their receptors. In addition to obesity, those with *BDNF* mutations also have impaired cognitive function, delayed developmental milestones, and hyperactivity. Those with loss of function of the TrkB receptor also have memory deficits.

*Lesson 2: Genetically Mediated Differences in Satiety Are Likely to Underly the Difference in Body Weight Seen in the Current Obesogenic Environment.* Defects in single genes causing severe non-syndromic human obesity result mainly through impacts on appetite. The recent rapid rise in obesity epidemic is clearly linked with the technological and social advances which have reduced the need for strenuous physical activity at work and at home, with abundant cheap, high-density food propagated with aggressive advertising. Nonetheless, the persistence of lean people in the current obesogenic environment suggests that either these people are able to withstand the toxic environment through conscious choices about diet and exercise and/or that they may have a genetic predisposition to stronger satiety signals, which enable them to achieve their leanness largely through unconscious mechanisms [30]. Already several common genetic variants predisposing to common obesity have been identified and appear to affect the same processes as monogenic causes of obesity (albeit of small effect size), supporting the latter explanation [7, 8].

## 3. Monogenic Pancreatic Beta Cell Diabetes

*Lesson 3: Proof of Key Components of Pancreatic Beta Cell Function and Responsiveness of Certain Genetic Etiologies to Oral Glucose Lowering Drugs Acting Distal to the Monogenic Defect.* The beta-cell monogenic diabetes are characterised by genetic mutations that result in early onset diabetes in absence of obesity or insulin resistance [31–33]. Most of the earlier mutations were identified via the candidate gene approach, that is, the selection of genes known to play a significant role in beta cell function and the subsequent demonstration of critical beta cell dysfunction when a mutation is present both under laboratory conditions and in humans who harbour mutations. More recently, novel and unexpected mutations that result in beta cell dysfunction have been demonstrated and have shed further light on normal beta cell physiology [34]. 

**Figure 2:**
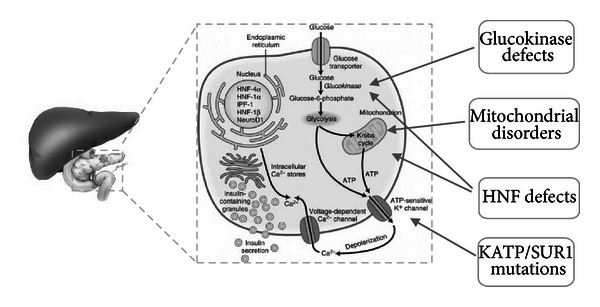
Location of common monogenic pancreatic beta cell defects leading to early onset of diabetes in absence of obesity-related insulin resistance. Hepatocyte nuclear factor (HNF), potassium adenosine triphosphate (KATP) channel, sulphonylurea receptor (SUR).

The beta cell is the major component of the pancreatic islets of Langerhans, which collectively account for 1-2% of the total pancreatic mass. The normal human beta cell has 3 functions that are critical to achieve its contribution to maintaining normoglycaemia. Firstly, the cell must be able to “sense” ambient glucose levels thus allowing any insulin output to be appropriate to requirements; secondly, the beta cell must be able to manufacture and store insulin; and thirdly, the cell must be able to rapidly secrete this insulin when required. Dysfunction of any of these roles will result in impairments of glucose homeostasis (see Figure 2). Typically, the mutations that result in manifestation of diabetes in early life, that is, neonatal diabetes mellitus (NDM), may be interpreted as more intrinsic to this process than those that are manifested later in life; however, the genotype-phenotype relationship for most of the pancreatic beta cell mutations are still unclear. 

### 3.1. Neonatal Diabetes Mellitus (NDM)

NDM refers to diabetes diagnosed in the first 6 months of life, which can be transient (approximately 70% of cases) or permanent, and is most commonly the result of a monogenic mutation [9, 35–37]. The incidence of permanent NDM (PNDM) has recently been calculated at 1 in 260,000 live births from a European registry, suggesting that the overall incidence of neonatal diabetes is considerably higher than previously recognized [37, 38]. Transient neonatal diabetes (TNDM) presents in the first weeks of life, remits spontaneously within 1–18 months, and may relapse to permanent diabetes during adolescence or early adulthood [9, 39, 40]. Genetic defects within the imprinted chromosomal 6q24 region are identified in the majority of cases of TNDM, with segmental paternal uniparental disomy, paternally inherited duplication of chromosome 6q24, or loss of methylation in the maternal copy of 6q24 seen. Overexpression of two paternally expressed genes: hydatidiform mole associated and imprinted (*HYMA1, *involved in mRNA encoding) and pleomorphic adenoma gene like 1 (*PLAGL1, *regulating cellular apoptosis) can be identified in 80% of these patients [41]. 

Activating mutations of the K_ATP_ channel genes (either potassium inwardly-rectifying channel, subfamily J, member 11 (*KCNJ11),* or ATP-binding cassette transporter subfamily C member 8 (*ABCC8*)) or insulin gene (*INS*) are the most common causes of permanent neonatal diabetes mellitus (PNDM), whilst *KCNJ11* and *ABCC8* mutations have been shown to account for a minority of cases of TNDM [35, 40, 42, 43]. The K_ATP_ channels link the ATP produced by cellular glucose metabolism to potassium flux and thus membrane excitability [44]. *KCNJ11* and *ABCC8* mutations cause an increase in the opening of the K_ATP_ channel resulting in hyperpolarisation, preventing beta cell membrane depolarization and insulin release [35, 42, 45]. Oral sulphonylurea (SU) drugs (such as glipizide, gliclazide, and glibenclamide) have long been used to treat type 2 diabetes and act through specific binding to the sulphonylurea receptor (SUR1) located on the K_ATP_ channel. The resultant closure of these channels stimulates insulin secretion, thus bypassing the effect of the *KCNJ11* and *ABCC8* mutations. Further validation of the function of the K_ATP_ channel is provided by the excellent response of many patients harbouring *KCNJ11/ABCC8* mutations to SU therapy; indeed, it is now standard practice for all patients with diabetes diagnosed before 6 months of age to undergo diagnostic testing for mutations in these two genes, with attempts to transfer those with confirmed mutations from insulin therapy to oral SUs [9, 46]. Furthermore, in those patients with K_ATP_ channel dysfunction evident in extrapancreatic structures (brain and muscle, as seen in cases of DEND (Developmental delay, Epilepsy, and Neonatal Diabetes syndrome)) the use of a nonbeta cell selective SU (glibenclamide) is also effective in treating the neuromuscular phenotype [46–48]. Cases of NDM caused by *INS* gene mutations do not respond to SU medications and currently require lifelong insulin therapy.

The reason why certain mutations within the K_ATP_ channel give rise to TNDM while others result in PNDM is unknown, but changes in pancreatic beta cell turnover or compensation at the level of the beta cell or whole body that is able to overcome the effects of these genes may be possible explanations [49].

Several more rare genetic etiologies of NDM have been identified, most of which are autosomal recessively inherited and are associated with extrapancreatic manifestations. These include pancreas-specific transcription factor 1A (*PTF1A,* pancreatic and cerebellar agenesis) [50], insulin promoter factor 1 (*IPF1, *pancreatic agenesis) [51], GLIS family zinc finger 3 (*GLIS3*, congenital hypothyroidism, glaucoma, kidney cysts and hepatic fibrosis) [52], regulatory factor X6 (*RFX6, *pancreatic hypoplasia, intestinal atresia and gall bladder hypoplasia) [53], GATA-binding factor 6 (*GATA6*, congenital heart defects) [54], neurogenin 3 (*NEUROG3, *congenital malabsorptive diarrhea) [55], neurogenic differentiation 1 (*NEUROD1, *sensorineural deafness and visual impairment) [56], forkhead box P3 (*FOXP3, *enteropathy and dermatitis) [57], eukaryotic translation initiation factor 2-alpha kinase 3 (*EIF2AK3*, exocrine pancreatic dysfunction, spondyloepiphyseal dysplasia, developmental delay, acute liver failure, osteopenia and hypothyroidism) [58], solute carrier family 2 (*SLC2A2, *aminoaciduria, proteinuria, short stature and rickets) [59], and solute carrier family 19 member 2 (*SLC19A2, *thiamine responsive megaloblastic anemia) [60]. 

The majority of infants with neonatal diabetes of diverse genetic etiologies are born with reduced birth weight, resulting from lower fetal insulin production, as fetal insulin is a major growth factor in utero [61].

### 3.2. Glucokinase (GCK) Mutations

Glucokinase (GCK) is a hexokinase enzyme that is found in the beta cell, liver, brain, and multiple other tissues in most mammalian species. GCK has many roles within expressing tissues but functions primarily as an ambient glucose sensor for the pancreatic beta cell [62]. The enzyme facilitates the conversion of glucose to glucose-6-phosphate at glucose levels of above 4.0 mmol/L, with adenosine triphosphate (ATP) produced as a secondary product. The increase in intracellular ATP initiates a cascade of membrane depolarisation that results in the extracellular release of preformed insulin containing granules [63]. Homozygous or compound heterozygous inactivating mutations in the *GCK* gene result in a severe diabetes phenotype, presenting at birth as permanent neonatal diabetes mellitus [64, 65]. Heterozygous, inactivating *GCK* mutations result in a higher concentration of glucose being required to stimulate use of the substrate and therefore insulin secretion. However, once this threshold is reached, insulin secretion therafter is relatively normal. Humans who harbour such heterozygous inactivating *GCK* mutations therefore demonstrate mild fasting hyperglycaemia but normal post meal time glucose levels [9]. Those who harbour heterozygous activating *GCK* mutations have the opposite clinical syndrome of hyperinsulinemic hypoglycaemia [66].

The population prevalence of heterozygous inactivating *GCK* mutations is approximately 0.1%, accounting for 20–50% of all cases of monogenic diabetes [67]. Mild fasting hyperglycaemia (5.5–8.0 mmol/L) is present from birth, whilst insulin response, and therefore postprandial glucose levels, are normal such that most patients demonstrate only small glucose excursions after meals (<3.0 mmol/L following a 75 g oral glucose tolerance test) [68]. Furthermore, there is no deterioration in the fasting hyperglycaemia with age, the HbA1c is normal or mildly elevated, and microvascular and macrovascular complications associated with other diabetes subtypes are not seen in persons harbouring *GCK* mutations [9, 69]. For this reason, ownership of a *GCK* mutation resulting in mild hyperglycaemia does not require any glucose lowering therapy.

*Lesson 4: Glucose Toxicity Is Not Seen in Those with Lifelong Mild Hyperglycaemia Resulting from Heterozygous GCK Mutations. *The glucose toxicity theory proposes that continual exposure to modest increases in blood glucose over a long period of time could has adverse effects on beta cell glucose sensitivity and function [70, 71]. No difference in the deterioration in glucose sensitivity with age has been found among GCK mutation carriers with lifelong mild hyperglycaemia compared with normoglycaemic controls [10]. This is in keeping with the stable glycaemia seen on prolonged followup of patients with heterozygous mutation and the modest decline with age seen in cross-sectional studies of patients with GCK mutations [68, 72]. Continuous exposure to the level of hyperglycaemia experienced by *GCK* mutation carriers has no significant progressive glucose toxic effect on the beta cell.

Up to 17.8% of pregnant women are diagnosed with gestational diabetes on the basis of recent guidelines, and up to 10% of these women will have a *GCK* mutation [73, 74]. However, the importance of suspecting and diagnosing a *GCK* mutation is high in pregnancy; treatment for “hyperglycaemia” in a pregnant woman with a *GCK* mutation may result in reduced birthweight if the foetus has inherited the mutation [75]. Conversely, if the foetus does not inherit the mutation, foetal hyperinsulinemia with resultant increased foetal growth, as a consequence of ambient hyperglycaemia in utero may occur, unless maternal blood glucose is reduced by insulin therapy. As the likelihood of inheriting the mutation is 50%, and prenatal genetic diagnosis is currently too invasive to perform solely for clarifying *GCK *mutation status, current advice is to perform serial ultrasonography to monitor foetal growth with consideration of treatment only in those with accelerated growth (i.e., those carrying babies presumed not to have inherited the GCK mutation) [74, 76].

*Lesson 5: Exposure to Mild Hyperglycaemia in Utero Does Not Program Offspring to Have Reduced Beta Cell Function. *There is animal and human evidence for maternal hyperglycaemia exposure in utero programming offspring to have beta cell dysfunction [77], however, these studies are confounded by polygenic predisposition to type 2 diabetes in such offspring. Non-mutation carrying offspring of mothers carrying the heterozygous inactivating GCK mutation was a good human model for studying the impact of exposure to hyperglycaemia in utero on offspring beta cell function. Such offspring had marked increase in birthweight as a result of being exposed to increased glycemia in utero but, despite this, had no evidence of altered beta cell function or glucose intolerance as adults, suggesting a lack of detrimental impact of hyperglycaemia exposure in utero in those without genetic predisposition to type 2 diabetes [11].

The significance of GCK in sensing ambient glucose has led to the hypothesis that pharmaceutical manipulation of this physiological mechanism in the form of glucokinase activators could present an effective method of treating hyperglycaemia as a result of type 2 diabetes [63]. No additional response of incretin hormone stimulation is likely from such GCK activators, as GCK is not the gut glucose sensor by which incretin cells release incretin hormones [10] as was previously hypothesized [78]. 

### 3.3. Hepatocyte Nuclear Factor (HNF) Mutations

The hepatocyte nuclear factor (HNF) family of proteins are ubiquitous and function primarily as transcription factors. Whilst predominantly expressed within the liver, their key role in regulating both pancreatic beta cell growth and function is demonstrated by mutations leading to progressive beta cell failure and childhood or early-adult onset of diabetes [79, 80]. 

Mutations in *HNF1A* are the most commonly encountered form of monogenic diabetes with a minimum prevalence of 50–60 cases per million population (accounting for 52% of all cases of monogenic diabetes) or 1-2% of all patients with diabetes [81]. Patients present in the second or third decade, although marked variability in the clinical phenotype (age of onset of diabetes, presenting features) are seen and relate in part to the type of mutation inherited [82]. Penetrance increases with age such that approximately two thirds of *HNF1A* mutation carriers will have diabetes by the age of 25, and >95% by the age of 40 [9]. Initially, insulin secretion at lower glucose levels is preserved, producing a characteristic glycaemic pattern with a normal fasting glucose and large excursion (>4.5 mmol/L) following a 75 g oral glucose challenge [83]. Progressive beta cell failure over time, however, leads to generalised hyperglycaemia, and unlike *GCK*-related monogenic diabetes, the risk of micro- and macrovascular complications appears to be comparable to type 1 and type 2 diabetes and is directly related to glycaemic control [84]. Patients with *HNF1A* mutations have an increased all-cause and cardiovascular-specific mortality rate when compared with nonaffected relatives (hazard ratios 1.9 and 2.3 resp.) [85]. The risk of coronary heart disease appears to be greater than that seen in type 1 diabetes which appears paradoxical when one considers the elevated high-density lipoprotein levels observed in those with *HNF1A* mutations (traditionally considered cardioprotective) [86]. Patients with *HNF1A* mutations frequently demonstrate high glucose lowering sensitivity to SU therapy [87–89]. As noted previously, SUs stimulate insulin secretion by binding directly to the beta cell membrane K_ATP_ receptor, thereby bypassing the metabolic pathways is rendered dysfunctional by an *HNF* mutation [87]. Consequently, patients misdiagnosed as type 1 diabetes and treated with insulin can be switched to oral SU therapy once the genetic diagnosis of *HNF1A* diabetes is made.

*HNF1A* directly regulates renal tubular expression of the sodium-glucose cotransporter (SGLT); HNF1A mutations consequently result in a low renal threshold for glucose (i.e, glycosuria despite normal or only slightly elevated blood glucose levels) as a result of impaired tubular glucose reabsorption [90]. This clinical feature often predates the onset of overt diabetes and can therefore be useful as a screening tool [83]. Additionally, HNF1A-binding sites are located at promoter sites in the gene coding for C-reactive peptide (CRP). Consequently, markedly lower high sensitivity CRP (hsCRP) levels are seen in monogenic diabetes as a result of an HNF1A mutation than in other forms of diabetes [91]. An hsCRP value of <0.4 mg/L has a sensitivity of 71% and specificity of 77% for diagnosing HNF1A (as opposed to type 2 diabetes) once type 1 diabetes and glucokinase mutations have been excluded. Furthermore, a urinary c-peptide to creatinine ratio appears useful in distinguishing HNF*1A/4A* monogenic diabetes from type 1 diabetes when diabetes has been present for many years given that C-peptide progressively declines in type 1 diabetes but is retained in HNF*1A/4A* monogenic diabetes [92].

Heterozygous inheritance of an *HNF4A* mutation results in a similar phenotype to the patients with *HNF1A* mutations and accounts for approximately 5–10% of all cases of monogenic diabetes. As with HNF1A mutation carriers, SU therapy remains an effective therapeutic strategy for these patients, but the elevated high density lipoprotein (HDL) levels and glycosuria are not seen in those with HNF4A mutations [93]. Somewhat paradoxically, hyperinsulinaemic hypoglycaemia has been documented in neonates with heterozygous HNF4A mutations, with resultant macrosomia (average increased birth weight of approximately 800 g). Consideration of genetic testing for HNF4A should therefore be considered in patients with clinical features suggestive of HNF1A monogenic diabetes but negative genotyping and those with a positive family history of macrosomia or neonatal hypoglycaemia. 

In contrast, mutations in *HNF1B*, which account for approximately 5% of cases of monogenic diabetes, lead to clinical syndromes more reflective of the widespread expression of this transcription factor [94]. Most carriers of this mutation appear to develop renal disease (cysts and dysplasia, glomerulocystic kidney disease, familial juvenile hyperuricemic nephropathy, and single kidney) which is the predominant clinical feature of this genotype. Indeed, only approximately 50% also develop diabetes. Unlike, HNF1A and HNF4A monogenic diabetes, pancreatic atrophy is common in those with HNF1B mutations, therefore carriers do not display the same sensitivity to SU therapy and often require insulin therapy. Other associated features include genital tract abnormalities, gout, biliary tree abnormalities and abnormal liver function tests. 

### 3.4. Mitochondrial Disorders Associated with Diabetes

Mitochondria are energy-generating organelles identifiable in most human cells [95]. Each mitochondrion, many thousands of which may be found within a single cell, contains abundant copies of mitochondrial DNA (mtDNA) which encode for components of mitochondrial oxidative phosphorylation and respiration. Maternal oocytes contain multiple mitochondria, whereas spermatozoa contain only a few hundred, most of which are destroyed during fertilisation. Thus, mitochondrial DNA, and any mutations contained within, can only be inherited maternally.

Mitochondrial DNA mutations can result in a wide range of multiorgan dysfunction with a number of recognised syndromes. Maternally inherited diabetes and deafness (MIDD) is the most commonly encountered mitochondrial diabetes syndrome and accounts for up to 1% of diabetes [96]. The syndrome is defined by the presence of diabetes and deafness with an inheritance pattern consistent with a mitochondrial disorder and is most commonly the result of a point mutation (*m.3243A*>*G*) [97]. These defects impair cellular energy generation, and therefore organs that are more metabolically active more frequently display clinical dysfunction. The endocrine pancreas is one such organ, thus diabetes is seen commonly in those with mitochondrial disorders generally. Beta cell failure with deficient insulin production is the characteristic feature, whilst insulin resistance is only seen in the presence of classical risk factors [98]. Most of the patients with MIDD display an insidious onset of glycaemic dysfunction, but occasionally more abrupt loss of beta cell function is seen. Indeed, one multicentre study demonstrated ketoacidosis as the presenting feature in 8% of carriers [99]. There is a wide age range of presentation of diabetes in carriers ranging from childhood to adulthood [96], and the penetrance for glycaemic dysfunction approaches 100% by the age of 70 years [100].

Deafness in MIDD is sensorineural in nature and results from defects in the cochlear and affects the majority of those with mitochondrial (m.*3243A*>G) diabetes [101]. Other systems that are frequently defective in MIDD include cerebrovascular (strokes, cerebral/cerebellar atrophy), opthalmological (macular dystrophy), cardiac (congestive cardiac failure), nephrological (focal glomerulosclerosis), muscular (myopathies), and endocrine systems (short stature secondary to hypothalamic dysfunction) [96]. Cellular heteroplasmy (i.e., a mixture of both functional and dysfunctional with respect to mitochondrial DNA) is likely to explain the significant variation in clinical phenotype in those who harbour these mutations [102].

*Lesson 6: Pancreatic Beta Cell Defects in Common Type 2 Diabetes Is Unknown but Is Likely to Be Multiple.* Monogenic pancreatic beta cell diabetes arising from key defects within the insulin secretory pathway results in diabetes without the confounding effects of obesity. Despite the contribution of obesity in type 2 diabetes, insufficient pancreatic beta cell function is the most likely cause. However, the site of the beta cell defect in type 2 diabetes is not known. Most common genetic variants identified as associated with type 2 diabetes are within genes important for beta cell function, beta cell development, or the regulation of beta cell mass [103] and overlap with some of the known causes of monogenic diabetes. Certain monogenic subtypes of diabetes have resulted in dramatic success of transferring from insulin therapy to oral sulfonylurea therapy, based on the location of the genetic defect upstream from the target of the sulfonylurea drug. If impaired beta cell regulation of K_ATP_ channel activity or glucokinase activity or HNF activity was the only problem in type 2 diabetes, then its phenotype would resemble that of patients with neonatal diabetes or *GCK* or HNF mutations, which it does not. Impaired mitochondrial metabolism has been postulated to contribute to type 2 diabetes, and an age dependent decline in mitochondrial function might explain why type 2 diabetes develops later in life. Collectively, the characteristics of the various monogenic defects and the temporary response to SU therapy observed in type 2 diabetes suggest that multiple processes downstream of mitochondrial metabolism and the K_ATP_ channel within the pancreatic beta cell, that regulate the amplification response to glucose or insulin exocytosis itself are most likely involved in the progression of type 2 diabetes [12]. The concurrent obesity-related insulin resistance is likely to place an increased functional demand on the beta cell and accelerate beta cell failure as is seen in cases of severe insulin resistance.

## 4. Monogenic Severe Insulin Resistance

Two groups of monogenic defects have been detected in patients with severe insulin resistance manifested in early life, (a) those that affect insulin signaling and (b) those that affect adipocyte development and/or function. 

### 4.1. Insulin Signalling Disruption

#### 4.1.1. Insulin Receptor Mutations

The most common group of patients with monogenic insulin resistance have a mutation in the insulin receptor (*INSR*), leading to a global reduction of insulin receptor function in target cells. Mutations in the *INSR* gene result in a clinical spectrum of disease severity ranging from mild to severe [13]. Less severe disease is usually caused by an autosomal dominant gene mutation in the *INSR* gene, in which patients present in the peri-pubertal stage or beyond with acanthosis nigricans, dysglycemia (either fasting hypoglycemia and postprandial hyperglycaemia or frank diabetes) in the presence of severe hyperinsulinemia, oligomenorrhea, and hyperandrogenism in women. In men, the presentation is less obvious with only acanthosis nigricans and sometimes fasting hypoglycaemia. Men often remain undiagnosed even after the development of diabetes requiring high doses of insulin. 

The most severe disorders, caused by rare, autosomal recessive mutations in the insulin receptor gene, result in almost complete lack of residual insulin receptor function. Such extreme insulin resistance is seen in patients with Donohue syndrome (or leprechaunism [104, 105]) and Rabson-Mendenhall syndrome [14]. Clinical manifestations include intrauterine and postnatal growth restriction, fasting hypoglycemia, postprandial hyperglycaemia, massive hyperinsulinemia, impaired muscle, and adipose tissue development. Death from intercurrent infection usually occurs in Donohue syndrome; however, Rabson-Mendenhall syndrome differs by the additional presence of dysplastic dentition, coarse facial features, severe diabetic ketoacidosis, pineal hyperplasia, and survival beyond infancy [106]. 

Patients with *INSR* mutation differ biochemically from patients with more common insulin resistance associated with the metabolic syndrome by having high levels of adiponectin, sex-hormone-binding globulin (SHBG), and insulin-like growth factor-binding protein 1 (IGFBP1) [107]. Adiponectin, SHBG- and IGFBP1 are all commonly reduced in people with obesity-related insulin resistance and various lipodystrophies, so these markers are helpful diagnostically in distinguishing those with insulin receptor dysfunction from those with other causes of severe insulin resistance.

*Lesson 7: Insulin Receptor Signalling on Pancreatic Islets Is Not Required for Beta Cell Compensatory Response to Severe Insulin Resistance.* The severe hyperinsulinemia seen even in those with almost complete loss of insulin receptor function suggests that the insulin receptor on pancreatic islets is not necessary for beta cell functioning, as has been suggested by some studies in mice [108]. However, the pancreatic beta cells eventually fail to compensate for severe insulin resistance and diabetes that follows over a variable period of time from a few days to beyond 40 years of age [13].

**Figure 3:**
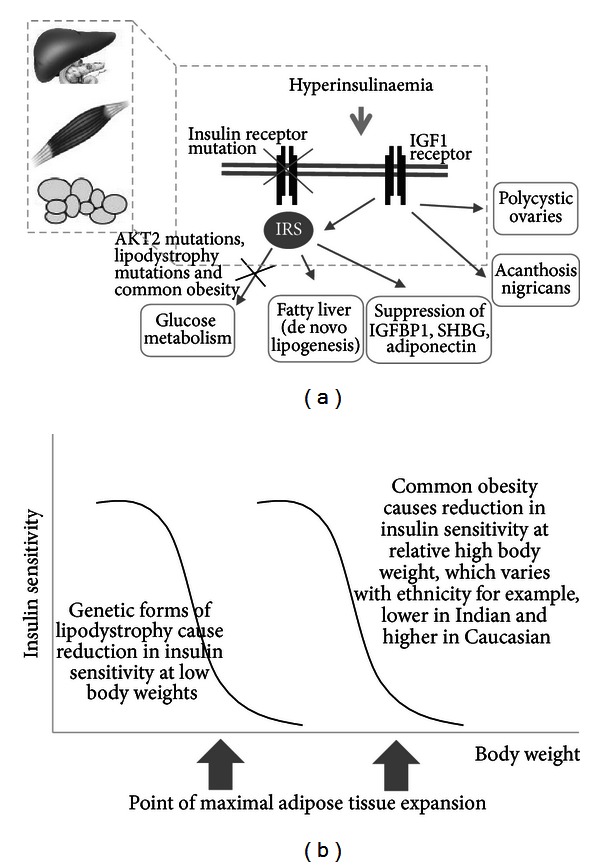
(a) Model of partial insulin resistance within different tissues highlighted by those carrying the insulin receptor mutation who have very high levels of circulating insulin which is able to bind to the insulin-like growth factor-1 (IGF1) receptor and stimulate the development of polycystic ovaries and acanthosis nigricans. In contrast to those with AKT2 (v-akt murine thymoma viral oncogene homolog (2) mutations and those with common obesity-related insulin resistance, those with insulin receptor mutations do not have fatty liver or suppression of insulin-like growth factor binding protein 1 (IGFBP1) or sex hormone binding globulin (SHBG) or adiponectin, which most likely requires active signaling through the nonglucose metabolism arms of the insulin receptor substrate (IRS) downstream pathway. (b) Tissue expandability theory model represents the individual set point up to which adipose tissue can be expanded without metabolic morbidity, which is likely to depend on genetic factors. The two curves represent the relationship of increasing body weight to reducing insulin sensitivity; however, the curve on the left represents the extremely limited adipose expandability in those who have a genetic mutation causing lipodystrophy, while those who have common obesity-associated reduction in insulin sensitivity have a more rightward shifted curve.

*Lesson 8: Acanthosis Nigricans and Ovarian Hyperandrogenism Are Likely to Be Mediated by Hyperinsulinemia Acting through Non-Insulin Receptor Pathways.* Acanthosis nigricans (a velvety hyperpigmentation of the skin) and ovarian hyperandrogenism (manifesting as hirsutism, oligomenorrhea, and polycystic ovaries in women) are seen both in patients with severe insulin receptor mutations who have reduced activity of the insulin receptor as well as in those with common obesity-related insulin resistance. This suggests that these clinical features are likely to be mediated by insulin signalling through a noninsulin receptor pathway such as by stimulating the insulin-like growth factor-1 (IGF-1) receptor [109]. Insulin is known to be capable of binding IGF receptors (although at lower affinity than to the insulin receptor) so when present in high concentrations it may bind and activate these receptors which have been found in skin and ovaries [14, 110] (see Figure 3(a)).

*Lesson 9: Development of Fatty Liver and Dyslipidemia Is Dependent on Adequate Insulin-Receptor Signalling. *Despite severe insulin resistance and compensatory hyperinsulinemia, patients with insulin receptor mutations appear to be protected from fatty liver and atherogenic lipid pattern [15]. This finding suggests that insulin action through the downstream insulin receptor pathway is required to produce these elements of insulin resistance, commonly observed in patients with highly prevalent obesity-related insulin resistance and hyperinsulinemia.

#### 4.1.2. AKT2 Mutations

Surprisingly few mutations have been identified in genes encoding the multiple downstream components of the insulin signaling pathway. A mutation in v-akt murine thymoma viral oncogene homolog 2 (*AKT2)* has been identified accounting for the clinical features of acanthosis nigricans, ovarian hyperandrogenism, diabetes mellitus, and in contrast to those with insulin receptor mutations, both metabolic dyslipidemia and fatty liver in the proband [111]. In addition, the presence of partial lipodystrophy seen in *AKT2* highlights the importance of *AKT2* in adipocyte differentiation as well as its role in the direct effects of insulin signaling. 

The presence of metabolic dyslipidemia and fatty liver in those with *AKT2 *mutations but not in those with an insulin receptor mutation suggests that the pathway required for hyperinsulinemia to drive liver fat accumulation and atherogenic very low-density lipoprotein (VLDL) secretion depends on the insulin receptor but not on AKT2. This supports the concept of selective postreceptor insulin resistance, also relevant to the highly prevalent form of insulin resistance [15] (see Figure 3(a)).

*Lesson 10: Selective Postreceptor (Partial) Hepatic Insulin Resistance Occurs in Common Metabolic Dyslipidemia rather than Total Postreceptor Insulin Resistance.* Although hyperinsulinemia in the context of normal blood glucose is often thought to be the hallmark of insulin resistance, the possibility of partial insulin resistance was not clear until studies of monogenic cases of insulin resistance were examined for the effect of hyperinsulinemia on actions such as suppressing hepatic lipogenesis [15]. It has been shown that the lipid metabolism in patients with primary lipodystrophy, AKT2 mutations, or severe insulin resistance of unknown etiology have exaggerated forms of metabolic dyslipidemia and fatty liver in contrast to those with INSR mutations who did not [15]. Those who had INSR mutations were found to have normal hepatic de novo lipogenesis in association with normal lipid profiles, while those with AKT2 mutations or lipodystrophy had elevated de novo lipogenesis and liver fat suggesting that reduced liver fat synthesis plays a key role in the protection from dyslipidemia in patients with insulin receptor mutations. It also supports that the signaling pathway responsible for insulin activation of hepatic de novo lipogenesis diverges from the insulin activation of glucose disposal pathway proximal to AKT2 activation, which is consistent with observations in mice [112] (see Figure 3(a)).

### 4.2. Monogenic Lipodystrophies

Lipodystrophy refers to heterogeneous disorders characterized by loss of body fat either from discrete or generalized areas, ranging from severe to mild. The molecular genetic cause of many types of inherited lipodystrophies has recently been discovered. 

#### 4.2.1. Congenital Generalized Lipodystrophy

Congenital generalized lipodystrophy (CGL) patients are recognized very early in life due to their near-total lack of body fat and strikingly muscular appearance [113]. Despite lack of body fat, diabetes, acanthosis nigricans, severe dyslipidemia (hypertriglyceridemia, and low high-density lipoprotein cholesterol), and fatty liver develop commonly in the teenage years or later. Most women have polycystic ovaries with impaired ovulation and fertility [114, 115].

Autosomal recessive (either homozygous or compound heterozygous) mutations in several genes have been implicated in the pathogenesis of CGL. Mutations in two genes: 1-acylglycerol-3-phosphate-O-acyltransferase 2 (*AGPAT2) *and Berardinelli-Seip congenital lipodystrophy 2 (*BSCL2)* account for the majority of cases and were identified through linkage analysis with positional cloning in affected families [114, 115]. *AGPAT2* is highly expressed in the adipose tissue and is one of the critical enzymes involved in the biosynthesis of triglyceride and phospholipids from glycerol-3-phosphate [116]. *BSCL2* is highly expressed in the brain and adipose tissue and its protein seipin is thought to be involved in lipid droplet formation and adipocyte differentiation [117, 118]. Patients with both *AGPAT2 *and *BSCL2* mutation have similar metabolic abnormalities; however, there are certain distinguishing phenotypic differences. Higher prevalence of mental retardation and cardiomyopathy is seen patients with *BSCL2 *mutations, and the presence of lytic lesions in the appendicular skeleton in patients with *AGPAT2* likely reflects the altered tissue expression and function of the two gene products [119]. 

Autosomal recessive mutations in polymerase 1 and transcript release factor (*PTRF)* [120, 121] and caveolin 1 (*CAV1)* [122] have also been identified in much rarer CGL cases. *PTRF* is involved in the biogenesis of caveolae which are invaginations of the plasma membrane found in many cell types but particularly abundant in adipocytes, muscle, and endothelia. *PTRF* regulates expression of caveolins 1 and 3, which are proteins found within caveolae, that are required for caveolae formation within many tissues [120]. *CAV1* is an integral component of caveolae, which binds fatty acids and translocates them to lipid droplets [123].

Patients with *BSCL2* mutations have the most severe loss of body fat including loss of fat from mechanical sites such as retroorbital, palm, sole, and periarticular regions, in addition to the metabolically active adipose tissue in the subcutaneous, intra-abdominal, intrathoracic, and other regions. However, those with *AGPAT2, CAV1,* and *PTRF* mutations do not have loss of mechanical fat. Both *PTRF* and *CAV1* mutation carriers have preserved bone marrow fat, which is absent in those with *AGPAT2* and *BSCL2* mutations [124].

*Lesson 11: Not All Fat Is Bad. *Given that obesity is associated with insulin resistance both longitudinally within individuals and cross-sectionally between individuals, this suggests that adipose tissue itself causes insulin resistance. However, the exaggerated appearance of insulin resistance features in rare patients with congenital lack of adipose tissue suggests that a normal amount of adipose tissue is critical for metabolic health. The different adipocyte-related cellular functions of the various congenital generalized lipodystrophy genes suggest that the common severe insulin resistance phenotype (diabetes, dyslipidemia, fatty liver) seen in these disorders is a result of the loss of adipose tissue rather than their specific gene functions in other tissues [16].

#### 4.2.2. Familial Partial Lipodystrophy

The first gene identified as causative of familial partial lipodystrophy (FPL) of the Dunnigan variety was lamin A/C (*LMNA) gene *[125], which remains the most common cause of FPL. *LMNA* encodes the protein lamin A/C which forms part of the nuclear lamina, and mutations have been linked to many other disorders such as Charcot-Marie-Tooth neuropathy, progeria syndromes, restrictive dermopathy, limb-girdle muscular dystrophy, and mandibulo-acral dysplasia [126]. The second gene to be identified in FPL was in peroxisome proliferator-activated receptor gamma (*PPARG)*, which is a nuclear hormone receptor most highly expressed in adipose tissue [127]. It is important in adipocyte differentiation and the target of thiazolidinediones class of diabetes medications. Since then, further genes have been identified including *AKT2 *[111], cell-death inducing DFFA-like effector c (*CIDEC)* [128], and perilipin 1 (*PLIN1) *[129]. Both *CIDEC* and *PLIN1* are important in lipid droplet dynamics and regulate triglyceride mobilization.

In all these FPLs, the failure of fat development is often not manifested until puberty and women are much more severely affected than men. This may relate to the higher adiposity seen in females than males, particularly with the onset of puberty. The phenotypic heterogeneity between the various genetic types of FPL is not clear; however, the partial loss of subcutaneous fat from the extremities is seen in all types. In the LMNA variety, the trunk is also affected but face and neck are spared, while there is preserved abdominal fat in the remaining types [124]. Despite this heterogeneity in truncal sparing of fat and overall less loss of body fat compared to CGL, the metabolic derangements in terms of diabetes, fatty liver, and dyslipidemia are similar among the different forms of FPL and comparable to that observed in CGL, yet more severe than that observed with common obesity-related metabolic syndromes. 

The lipodystrophies suggest that there is a critical amount of adipose tissue required for buffering episodic excess caloric intake by serving as a sump for free fatty acids and glucose. Loss of this capacity produces increased uptake in other metabolically less suited tissues such as liver, muscle, and pancreatic beta cells where harmful effects include fatty liver, dyslipidemia, and loss of insulin sensitivity and insulin secretion. An exacerbating factor may include the loss of adipocyte-tissue-derived leptin which signals adequacy of adipose tissue to the brain. Loss of leptin in CGL (and to a less extent FPL) suggests that whole body energy stores are low and leads to compensatory hyperphagia. The resulting increase in ingested calories worsens systemic metabolic stress. Proof of this has been illustrated by the dramatic efficacy of leptin replacement in patients with most lipodystrophies [130, 131].

The rare lipodystrophy syndromes support the adipose expandability hypothesis [132, 133]. This proposes that individuals have a fixed capacity for adipose tissue expansion to cope with excess energy consumption (very low in those with lipodystrophy but very high in those with morbid obesity without associated features of metabolic syndrome). With chronic consumption of excess calories, this threshold is exceeded (quickly in those with lipodystrophy and more slowly in most people with common metabolic syndrome), and fat is forced to accumulate in other organs such as the liver, skeletal muscle and pancreatic beta cells where they result in fatty liver, dyslipidemia, insulin resistance, and diabetes [13] (see Figure 3(b)).

## 5. Conclusions

The observation that loss of function of certain genes in humans leads to either severe obesity, early diabetes, or severe insulin resistance (with or without lipodystrophy) is very powerful in illustrating how defects in specific encoded proteins located predominantly in the brain, pancreatic beta cell, muscle, and or fat give rise to these distinct components of the metabolic syndrome. They challenge the view that environmentally driven obesity leads to insulin resistance which leads to type 2 diabetes, as supported by many cross-sectional and longitudinal epidemiological studies. The monogenic disorders provide particular insights that (1) there are clear biological driven origins of obesity affecting appetite, which predisposes to insulin resistance and type 2 diabetes, (2) specific defects in pancreatic beta cell function predispose to diabetes in absence of either obesity or insulin resistance, and (3) specific defects in insulin signaling or fat storage capacity can lead to severe insulin resistance in absence of obesity, which leads to diabetes through pancreatic exhaustion. 

All single-gene defects discovered to be responsible for human obesity have been identified to impact on appetite [134]. Common genetic variants predisposing to obesity appear to affect the same processes [135]. This suggests that although the recent obesogenic environment has resulted in an overall tendency to gain weight in all, interindividual differences in susceptibility to this environment may have a biological explanation (likely through differences in satiety), rather than necessarily a moral explanation through conscious individual efforts to diet and exercise [30]. 

 Various monogenic pancreatic beta cell diabetes arising from key defects within the insulin secretory pathway results in diabetes, manifesting from early infancy to early adulthood. Certain monogenic subtypes of diabetes have resulted in dramatic success of transferring from insulin therapy to oral sulfonylurea therapy, based on the location of the genetic defect upstream from the target of the sulfonylurea drug. The confounding effects of obesity are less important in these disorders, so it is possible to translate some of these findings to the etiology of type 2 diabetes. Common genetic variants predisposing to type 2 diabetes have been identified in many beta cell targets overlapping with known causes of monogenic diabetes [103]. Current evidence favours that insufficient glucose-stimulated insulin secretion is primarily responsible for type 2 diabetes; however, the site of the beta cell defect in type 2 diabetes is not known. Collectively, the monogenic diabetes phenotypes and the temporary response of type 2 diabetes to SU therapy suggest that multiple processes downstream of mitochondrial metabolism and the K_ATP_ channel within the pancreatic beta cell, that regulate the amplification response to glucose or insulin exocytosis itself, is involved in type 2 diabetes [12]. The associated obesity-related insulin resistance in type 2 diabetes is likely to place an increased functional demand on the beta cell and accelerate beta cell failure, as is seen in cases of severe insulin resistance.

In contrast to insulin resistance commonly observed in relation to obesity, patients with monogenic defects resulting in severe insulin resistance occur in absence of obesity or in the presence of generalized or regional lack of adipose tissue. Such lipodystrophic patients from diverse genetic etiologies in fat metabolism have a defect of triglyceride storage in adipose tissue, which results in lipid accumulation elsewhere in the body. This leads to severe insulin resistance associated with the usual accompanying characteristics of fatty liver and atherogenic dyslipidemia. At a more direct level, those who have a defect in their insulin receptor have very high levels of insulin resistance and frequently develop diabetes from compensatory beta cell exhaustion, yet are protected from fatty liver and atherogenic dyslipidemia. Patients with insulin receptor defects also differ biochemically from other types of insulin resistance by displaying elevated adiponectin, IGFBP1, and SHBG, which are all commonly suppressed in common insulin resistance or lipodystrophy-associated severe insulin resistance. This highlights the mechanisms behind partial insulin resistance or postreceptor selective defect in signaling through one arm of the insulin signaling pathway evident in common obesity-related insulin resistance.

Several monogenic disorders manifesting the key components of the metabolic syndrome have enabled us to unravel the pathogenic link between the defined molecular defect (or groups of defects) and obesity, early diabetes, or severe insulin resistance, each without the confounding effects of all the other components. Moving forward, we should continue to discover additional monogenic causes for the remaining portion of patients with severe disorders who currently do not have any defined etiology, which will not only help improve their management but may uncover future novel and therapeutic targets for the increasingly common metabolic syndrome.

## Figures and Tables

**Table 1:** Summary of lessons learned from monogenic disorders resulting in nonsyndromic obesity, pancreatic beta cell diabetes, and severe insulin resistance.

	Lessons	Human examples	References
1	Proof that humans can become obese as a result of single-gene defects controlling key central components of appetite	Several etiologies of severe human obesity result from single genes involved in appetite pathways for example, *LEP*, *LEPR*, *POMC*, *MC4R*, *BDNF*, *TrkB *	[1–6]
2	Genetically mediated differences in satiety are likely to underly the difference in body weight seen in the current obesogenic environment	Several common single-nucleotide polymorphisms involving similar appetite components for example, *MC4R* and *BDNF* have been identified at greater frequency in those with common obesity	[7, 8]
3	Proof of key components of pancreatic beta cell function and responsiveness of certain genetic etiologies to oral glucose lowering drugs acting distal to the monogenic defect	Those with mutations in *KCNJ11, ABCC8, HNF1A, HNF4A* are able to be treated with sulphonylurea tablets rather than insulin, given that their molecular defects are upstream of the *SUR1* receptor where sulphonylureas act to promote insulin secretion	[9]
4	Glucose toxicity is not seen in those with lifelong, mild hyperglycaemia resulting from a heterozygous glucokinase mutation	Those with heterozygous *GCK* mutations have stable, mild hyperglycaemia with no deterioration in beta cell function with age	[10]
5	Exposure to mild hyperglycaemia in utero does not program non-mutation carrying offspring to have reduced beta cell function	Non-mutation carrying offspring born to mothers with *GCK* who have experienced mild hyperglycaemia in utero do not have reduced beta cell function compared to those born to fathers with *GCK *	[11]
6	Pancreatic beta cell defects in type 2 diabetes are likely to be multifocal including sites distal to the SUR1 receptor where sulphonylureas act to promote insulin secretion	The progressive failure of sulphonylurea therapy in those with type 2 diabetes compared to durable response seen in monogenic causes upstream of *SUR1* receptor	[12]
7	Insulin receptor signaling on pancreatic islets is not required for beta cell compensatory response to severe insulin resistance	Those with a global defect in their insulin receptor due to *INSR* mutations have dramatically high levels of circulating insulin	[13]
8	Acanthosis nigricans and ovarian hyperandrogenism are likely to be mediated by hyperinsulinemia acting through non-insulin receptor pathways	Those with a global defect in their insulin receptor due to *INSR* mutations have marked acanthosis nigricans and such women have ovarian hyperandrogenism	[14]
9	Development of fatty liver and dyslipidemia are dependent on adequate insulin-receptor signalling	Those with a global defect in their insulin receptor due to *INSR* mutations do not develop fatty liver or dyslipidemia, despite markedly elevated levels of circulating insulin	[15]
10	Selective postreceptor (partial) hepatic insulin resistance occurs in common metabolic dyslipidemia rather than total postreceptor insulin resistance	Fatty liver and dyslipidemia frequently coexist with common metabolic syndrome insulin resistance	[15]
11	Not all fat is bad	Those with inherited defects in fat metabolism resulting in partial or complete loss of body fat have exaggerated dyslipidemia, fatty liver, and insulin resistance	[16]
